# Supplementary material for: Are carbon emissions trading and green financial instruments synergistic? -Comprehensive quantitative research based on content analysis
Source: PLoS One. 2024 Mar 7;19(3):e0298601. doi: 10.1371/journal.pone.0298601 (PMC10919601; doi:10.1371/journal.pone.0298601)
Supplement: S1 Data — (ZIP) [file pone.0298601.s001.zip › Data/地级市Z_F工作报告.zip!/┴╔─■/╕o╦│╩╨/└┤╫╘╣1⁄2╓┌║┼í╛┬φ┐╦╩2╛▌═°í┐_2007.docx]

各位代表：

现在，我代表市人民政府向大会报告工作，请予审议，并请政协各位委员和其他列席人员提出意见。

一、2006年主要工作回顾

刚刚过去的一年，是全市经济社会快速发展的一年，是各项工作取得重大进展的一年。一年来，我们在市委的正确领导下，在市人大和市政协的监督支持下，团结带领全市人民，同心同德，务实苦干，全面完成了市十三届人大四次会议确定的主要目标任务，实现了“十一五”的高起点开局。预计实现地区生产总值457.5亿元，同比增长15.1%。固定资产投资201亿元，增长33.0%。社会消费品零售总额202亿元，增长13.8%。财政一般预算收入27.5亿元，一般预算支出59.6亿元，分别增长23.4%和17.4%。城市居民人均可支配收入9305元，农民人均纯收入4160元，分别增长16.2%和11.1%。万元生产总值能耗降低4%。

“开放年”成效显著，项目建设取得重大突破。加大走出去与请进来的力度，抚顺的开放氛围和对外知名度明显提升。全市外资总投入1.1亿美元,注册外资额5279万美元，分别增长18.9%和25.5%；STX等重点外资企业发展势头良好；域外引资项目683个，资金到位121.8亿元、增长156.4%，其中超亿元项目41个；外贸出口5.05亿美元，增长26%。年初确定的121项市级重点项目开工113项，完成投资119亿元，其中百万吨乙烯、电解铝环保改造、热电联供机组、大伙房水库输水工程等大项目相继开工建设。抚顺经济开发区先导示范作用日益增强，开工建设项目39个，其中投资超亿元项目16个，完成投资30.2亿元、增长4倍；抚顺高新区被批准为省级高新区。工业园区建设迅速推进，起步区大都达到了项目摆放条件，签约和在建项目11个，总投资18.4亿元。通过“开放年”的努力，一大批优势产业项目纷纷落地，抚顺人盼望多年、为之奋斗多年的大发展，正在变成现实。

国企改制攻坚基本完成，工业经济增速加快。以新抚钢股份制改造、抚顺铝厂资产重组并入中国铝业、红透山矿并入中国有色为标志,国企改制取得重大成果，全年完成国企改制105户，累计完成459户，基本完成改制攻坚任务；改制企业的活力迅速释放，成为拉动全市经济的重要增长点。工业经济的增速和后劲明显增强，规模以上工业增加值增长16.2%；完成工业投资80亿元、增长16%；开发新产品新技术420项、增长5.5%。品牌战略有效推进，昆仑牌全精炼石蜡成为我市第一个中国名牌产品。

新农村建设扎实推进，农村经济全面发展。积极推进农业产业化、规模化和标准化，新增产业化龙头企业16户，增长23%；新建标准化生产示范基地12个、5万亩；新发展农民专业合作经济组织168个。粮食产量58万吨，创历史最好水平。建立国家、省、市三级粮食储备体系，粮食安全保障得到强化。农村基础设施进一步完善，沈阳至南杂木高速公路建成通车；68个行政村铺设了油路；关山水库风景区建设稳步推进；修复和新建堤防695公里、新上农村自来水工程295处，复垦耕地2.1万亩，“8?13”水毁工程全部恢复并超过灾前水平，夺得省农田建设大禹杯已成定局。农村生态建设得到加强，植树造林14.3万亩，是计划的2.18倍；新开发林地21.5万亩，完成任务的165%；新建生物质气化工程14处、沼气池4000座，又有8000户农民用上了新能源。完成集体林产权制度改革320万亩，超额完成省定任务；健全城乡动物卫生监督网络，无重大动物疫情发生；整体推进30个村开发式扶贫，新建移民小区14个，3.5万农民脱贫。农业综合开发促进了农民增收和农村经济发展。

县区经济取得突破性进展，现代服务业快速发展。以实行分税制财政体制和下放国有企业为核心，形成了促进县区加快发展的激励机制，县区发展势头强劲，后劲显著增强，生产总值和固定资产投资增幅分别达27.7%和62.6%。旅游基础设施进一步完善，皇家极地海洋世界建成开馆，红河峡谷漂流晋升为国家4A级景区，满族风情国际旅游节成功举办，使我市成为东北地区具有较强吸引力的旅游目的地，对地区经济发展的拉动作用明显增强，全年实现旅游总收入44.8亿元，增长一倍以上。商贸流通业快速发展，新增连锁店32个，发展“农家店”180个，销售额超亿元的流通企业达到8家，全市商贸流通业新增税收7000万元、新增就业8000人。房地产开发施工168万平方米、竣工115万平方米；信息产业实现增加值9.6亿元，增长22%。民营经济发展迅猛，新增规模以上企业150户、销售收入超亿元企业10户；民营经济预计完成增加值171亿元，上缴税金18亿元，分别增长26.7%和38.5%，民营经济占全市经济比重达37.4%。

城市建设和管理力度加大，市区环境明显改善。坚持省投资、快速度、创精品，开展大规模的城市建设，共计改造道路101条、110万平方米，亮化“摸黑”路7条，特别是抚顺人民盼望多年的高山路改造和南环路建设顺利推进；和平桥、汪良桥、前甸通道、高阳通道等重点道桥建成使用，不仅改善了交通，而且为城市增加了几道靓丽风景；南站广场、站前商业区和劳动公园一期改造如期完成，深受市民欢迎；整治浑河城市段干支流河道18公里，改造和扩建3个水厂，改造“三供”管网76公里、排水管网31.1公里，绿化硬化居民小区46个、111万平方米。强化城市综合行政执法管理，依法拆除违法建筑物1230处、违章户外广告3580处，34个露天占路集贸市场被取缔、规范或实现退路进厅；完成永寿路综合停车场建设，实施南站地区公交线路调整和105路小客线路整治，新增、更新公交车辆188台，新建现代化候车亭94个，公交秩序和形象明显改善。全面开展城乡环境综合整治，重点实施了31个大气污染治理和20个畜禽养殖污染治理工程，整治1吨以下直燃原煤锅炉1300多台。植树造林150万株、新增绿地53公顷，城区空气质量达到二级以上标准的天数比上年增加12天，浑河、大伙房水库水质达到五类和保持二类水体标准。加强土地收购储备工作，全年收储336公顷，上市出让354公顷，实现收益6.5亿元。切实整顿和规范矿产资源开发秩序，严厉打击各种非法采矿行为，全市矿山企业由317家减至259家。作为老工业基地的抚顺，经过几年共同奋斗，生态环境和人居环境发生了令人瞩目的变化，路宽了、天蓝了、树绿了、水清了、景美了，抚顺人开始为拥有这样的城市而感到自豪，也激发和坚定了建设家乡的热情和信心。

民生工程扎实推进，社保体系不断完善。政府承诺为民办的10件实事全部落实，其中棚户区房屋拆迁51.9万平方米，开工建设安置用房201.4万平方米、竣工190万平方米，拆迁建设规模、推进速度和居民回迁率均居全省第一，并被国家开发银行推为经典项目。棚户区改造不仅根本改善了居民的生活条件，而且创造了“棚改精神”，成为加速抚顺振兴的强大精神力量。采沉区搬迁进展顺利，新建续安小区住宅19.5万平方米，安置居民2375户。解决龙凤等地区吃原水1万户、吃水难1.1万户、煤气拖期开栓435户，市区居民吃原水问题基本得到解决。颁布实施《抚顺市城市特困居民医疗救助暂行办法》，启动了门诊救助和住院救助相结合的城市医疗救助制度。筹集款物1716万元，救助困难群众7.2万户；颁布实施《抚顺市老年人权益保障条例》，为高龄老人办理了人身意外伤害保险。以零就业家庭、棚改回迁居民为重点，加大就业再就业工作力度，全市城镇实名制就业13.4万人、培训3.4万人、劳务输出3.8万人、1419户零就业家庭全部实现至少一人就业，棚改回迁下岗失业人员就业率达到85%以上，实现了棚改居民搬得回、住得稳。社保扩面征缴工作取得突破，共计征缴各项社保基金14.3亿元、增长25.1%，其中养老保险新参保企业823户、2.3万人，新参保人员缴费率100%，征缴企业养老保险统筹基金比上年增加1.4亿元，增长25.2%，达到历史最高水平。调整企业退休人员养老金工作进展顺利，城市低保标准提到180元，17.4万城市居民、2.5万农村居民被纳入最低生活保障，基本实现应保尽保。

社会事业协调发展，社会大局和谐稳定。先后与清华大学、中科院北京分院签订了全面合作协议，围绕资源深加工，加强与10余家科研院所的交流合作，中油抚顺石化公司、抚顺挖掘机厂成为博士后科研工作站和民企博士后科研基地，50余个科技项目得到国家和省支持。消灭农村D级危房校舍2.4万平方米，建成和在建九年一贯制学校8所，实现农村三县中小学现代远程教育全覆盖，改造薄弱学校43所，资助义务教育阶段贫困生2.1万人，帮助607名贫困生圆了大学梦，高考录取率高于全省平均水平11个百分点。医疗救治体系进一步完善，公共卫生体系初步形成，改造乡镇卫生院18所，新型农村合作医疗和社区卫生服务机构覆盖面均达100%，新型农村合作医疗参合率达77.5%。群众文化活动全面展开，广场演出超过2100场，涌现出一批国家和省级文化工作先进社区；满族秧歌进入国家第一批非物质文化遗产保护名录，成功举办了全国满族秧歌邀请赛，平顶山遗址纪念馆改扩建工程进展顺利。成功承办了辽宁省第六届少数民族传统体育运动会并创佳绩，省十运会金牌排名比上届提升4个位次，建成城乡体育健身场所80处，开展群众喜闻乐见的体育赛事和活动400多项次。基本完成有线电视村村通工程。人口出生率和自然增长率分别为5.56‰和0.39‰。加大平安工程建设投入，防控工作全面推进，打击犯罪能力明显增强，执法水平不断提升，实现了社会治安大局持续稳定，群众的安全感和满意度明显提高。加强监管体系建设，确保群众食品、药品安全。深入贯彻《信访条例》，实行联席会议制度，坚持领导包案、源头治理、专项治理、集中治理相结合，信访总量同比下降20.5%，进京、去省集体访明显回落。努力为民排忧解难，市长公开电话转办问题解决率达93.1%。此外，安全生产、消防、审计、统计、外事、宗教、物价、供销、人防、地震、档案、社会科学、双拥、妇女儿童、老年人、残疾人等工作也都取得了新成绩。

政府建设不断加强，发展软环境进一步改善。自觉接受市人大法律监督和政协民主监督，坚持定期报告工作；办理人大代表建议279件、政协提案509件，满意率达99.7%。政府工作更加规范化、程序化，全年完成立法13件，行政许可项目由246项削减为175项，非许可类行政审批事项由113项削减为64项，行政执法责任制纳入政府目标管理，依法行政能力明显提高。全面启动“五五”普法。大力推进政务公开，颁布实施抚顺市突发公共事件总体应急预案和20个专项预案及8个部门预案。实施“企业安宁日”制度，在全省率先开通“民心网”，公共行政服务中心建立重大投资项目快速审批“绿色通道”，进一步提高了行政效率。全面推进事业单位机构改革，减少事业单位130家，精简人员编制6300名。加强绩效考核和社会舆论监督，政府工作人员的服务意识和工作效率有所提高。

各位代表，过去的一年，是可圈可点、好事连连的一年，是人心齐、干劲足，亮点多、效果好的一年，是强本固基、蓄势待发的一年。我们完全有理由说，抚顺发展已经步入快车道，抚顺人民美好的愿望正在实现！我们所取得的成就，是省委、省政府和市委正确领导的结果，是抚顺人民辛勤工作的结果。在此，我代表市政府，向为抚顺发展做出贡献、付出艰辛的全市各族人民致以亲切的问候和崇高的敬意！向给予监督支持的人大代表、政协委员，各民主党派、工商联和无党派人士、人民团体，向参与全市发展建设的中央和省在抚单位、驻抚部队指战员、武警官兵和各界人士、海内外朋友表示衷心感谢！

在肯定成绩的同时也必须清醒地看到，我市经济社会发展中还存在着不少困难和问题，主要表现在：一是经济发展速度和效益还不高，调整优化结构和转变增长方式的任务还很艰巨，资源深加工尚未迈出大的步伐。二是生态环境治理任务繁重，能源资源浪费现象依然存在，科学发展观的要求还没有完全落到实处。三是财政收支矛盾仍然突出，刚性支出持续增加，养老保险基金缺口较大。四是一些群众关注的热点难点问题解决得还不尽如人意，特别是在就业、社保、就医、上学、治安、供热等方面，群众还有不少意见。五是一些政府部门和工作人员在思想观念、服务意识、工作作风和办事效率等方面，与加快发展和人民满意的要求差距较大，推诿扯皮、以权谋私的现象还时有发生。这些问题已经引起我们的高度重视，正在并将继续采取有效措施认真加以解决。

二、2007年主要工作

2007年是实施“十一五”规划的第二年，也是加快抚顺振兴的关键一年。面对国家加强和改善宏观调控，实现经济又好又快发展的良好外部环境；面对中央加大对东北老工业基地振兴支持力度，国内外投资商持续关注辽宁的有利条件；面对我市国企产权改革基本完成，一批大项目落地开工和工业园区顺利建设的坚实基础；面对全市上下顽强奋进、和衷共济、渴盼发展、比拼实干的浓厚氛围，我们对新的一年充满信心。只要我们统一思想，抓住机遇，苦干实干，就一定能够创造更加辉煌的工作业绩，早日实现抚顺老工业基地的振兴。

今年政府工作的总体要求是：以邓小平理论和“三个代表”重要思想为指导，全面落实科学发展观，以“项目年”为牵动，强力抓好重点项目推进和工业园区招商、社会主义新农村建设、城市功能完善和发展空间拓展、民生改善，着力推进经济结构调整和增长方式转变，确保社会和谐稳定，努力实现经济又好又快发展和社会全面进步。

全市经济社会发展主要预期指标：地区生产总值增长16%；全社会固定资产投资完成280亿元、增长39%；实际总投外资额1.3亿美元、增长18%，注册外资额6500万美元、增长23%，引进域外资金150亿元、增长23%，外贸出口5.4亿美元、增长7%；全地区财政一般预算收入30亿元、比上年实际增长9.1%，一般预算支出40.9亿元、比上年预算增长18.8%；全社会消费品零售总额230亿元，增长14%；城市居民人均可支配收入、农民人均纯收入分别增长16%和12%；万元生产总值能耗下降4.4%。

（一）扎实开展“项目年”活动，务求招商引资取得更大成效

举全市之力推进项目。要以抓项目促结构调整、环境改善、作风转变，推动经济社会快速发展。主要目标是实施重点项目120项，计划投资222亿元。加紧项目库建设，围绕地区优势和结构优化，认真做好各行各业项目的策划、筛选和包装。加大“上争下促、外引内培”的力度，上争即争取更多的项目纳入国家和省支持的范围，下促即促进各地区和部门一心一意抓项目、上项目，外引即强化招商引资，内培即培育和支持域内企业上项目。

再掀招商引资新高潮。继续坚持“境外、域外、域内”三资并重，最大限度地吸引外来项目和资金，“两区四园”的招商引资要实现大的突破。突出重点招商区域，加大走出去与请进来的力度，有针对性地对韩国、日本、欧美、东南亚、港澳台、长三角、珠三角、沈阳及周边城市等资本密集地区开展招商引资；采取小团组、专业化、多批次等方式，开展产业招商、园区招商、产业链招商、以商招商。突出招商队伍建设，培养一批懂规则、会运作、素质高的专业人才，提高招商引资的成功率。围绕资源深加工，招商引进项目1000个，其中投资超亿元项目100个；确保开工项目500个，其中投资超亿元项目50个；当年竣工项目300个，其中投资超亿元项目30个。抚顺经济开发区要充分发挥先导作用，实现域外招商引资额40亿元、增长32.5%。

加强服务和考核。建立健全领导联系重点项目制度、重点项目推进联席会议制度以及项目推进通报制度等，加强跟踪管理，及时协调解决问题；加强服务支持，在项目立项申报、环保评审、拆迁安置等环节提高效率，为项目顺利推进创造良好环境。全面落实目标责任制，将项目和招商引资工作作为评价各县区、相关部门的重要标准。

（二）坚持工业强市，壮大经济规模与实力

优化存量、巩固老优势。继续围绕做大做强石化、冶金、机械、电力、建材、电子等传统优势产业，加快用信息技术改造传统产业的步伐，大力支持其在调整结构和转变增长方式中实现规模扩张、水平提升。石化业重点推进中油抚顺两大公司实施“千万吨炼油、百万吨乙烯”项目，加快建设东北最大的石油炼化生产基地。冶金业重点推进铝业公司电解铝、海绵钛的扩能改造，打造全国最大的钛、镁系列生产基地和东北最大的铝系列生产基地；支持新钢铁实施搬迁和系列技术改造。装备制造业重点推进抚挖公司履带起重机改造、普利达公司煤矿防爆电机搬迁改造、石油机械大型余热锅炉改造、机械公司大型换热器改造等骨干项目，提升装备制造业的档次和规模。电力业重点推进抚电两台30万千瓦机组建设以及抚铝自备电厂、中胜、辽电新增供电机组的立项核准建设，使抚顺成为东北重要的电力供应基地。建材业重点扩大水泥等产品生产规模，围绕塑料和钢铝资源发展新型建材。信息产业重点扶持发展电子产品制造业，逐步把我市建成全国煤矿安全监测监控产品和辽宁省电视机生产基地。煤炭业重点推进抚矿集团加快战略转型，大力发展接续产业和非煤产业。

做大增量、打造新优势。围绕资源深加工和延长产业链，吸引新投资，鼓励支持已有企业发展壮大。一是加大“两区四园”建设及拓展力度，规范园区政策，严格园区准入，支持成长性强、投资密度大、投入产出率高的企业进驻园区；实施老城区改造的“退二进三”战略，鼓励城内的工业企业迁入工业园区，并实现技术升级和结构调整。二是继续深化国企改革，采取特殊政策和措施，争取盘活一批“空壳”企业；加强对改制企业的服务和支持，促其内在活力和潜能充分释放。三是扩大中省直企业与地方中小企业的协作配套，全年配套额达到30亿元、增长25%以上。四是鼓励企业开展科技创新，开发新产品400项，争创省级以上名牌产品10个、著名商标5个。五是加大综合协调力度，全力推进企业扭亏增盈，提高工业经济运行质量和效益。

（三）壮大县区经济实力，促进服务业和民营经济大发展

鼓励支持县区实现跨越式发展。巩固完善市与县区分税制财政体制，落实各项支持政策，赋予县区更大的自主权，调动县区加快发展的积极性。按照县区经济特色化、特色经济产业化、产业发展规模化的思路，积极培育发展县区特色主导产业，形成各具特色的县区经济格局。鼓励县区以园区和城镇为载体，大力发展特色优势工业和中小企业集群，培育发展一批拉动和支撑县区经济的利税大户。充分发挥城区带乡的优势，拓展城市发展空间。

实现民营经济发展新突破。加大扶持，营造氛围，推动全民创业。鼓励民营企业加大技改投入，重点支持专、精、特、新的科技型民营企业。引导民营企业创新管理机制，建立现代企业制度，迅速做大做强。认真落实各项扶持政策，加大服务力度，重点抓好中小企业融资担保体系建设，为中小企业发展创造良好的金融环境。民营经济增加值、税金分别增长27%和20%以上。

加快发展以旅游、商贸为重点的服务业。旅游业要整合现有资源，打好清前史迹、绿色生态、红色旅游等特色品牌，抓好皇家极地海洋世界二期、皇家百老汇一期、萨尔浒风景区等重点项目建设；挖掘文化内涵，完善基础设施，优化精品线路，做强特色产品，加大宣传策划和包装推介力度，办好2007中国（抚顺）满族风情国际旅游节和中外旅行商大会，提高抚顺旅游知名度，全年旅游接待人数、旅游总收入分别增长20%和22%。商贸流通业结合城区企业易地搬迁和危旧楼房改造，抓紧规划建设一批商务设施；加快南站地区商业时代广场、裕民服装城等重点项目建设，积极招驻国内外知名商家来抚开店；规划建设辐射面广、带动性强的专业批发市场和小商品市场，继续实施“万村千乡”工程，力争在农村新开连锁超市200家。加快通信基础设施建设，实现宽带进村入户。房地产业要以满足百姓需求为重点，保持适度规模，坚持有序开发，加大经济适用房建设，施工170万平方米、竣工120万平方米。

（四）以农民增收为核心，加快社会主义新农村建设

大力发展现代农业。坚持用工业化理念谋划发展农业，围绕食用菌、山野菜、中药材、有机大米和绒山羊、林蛙等优势产业和绿色资源，积极推进产业化经营。扶持和引进一批规模大、牵动力强的龙头项目，年内销售收入超亿元及5000万元以上的企业力争分别达到15户和30户；搞好生产技术推广及农产品检验检测，大力推行无公害、标准化生产，加强国家级标准化示范区建设；围绕优势产业，建设并扩大种养基地，争取完成中药材GAP产地认证品种3个。大力发展林地经济，林地开发20万亩。整合绿色农业资源品牌，统一打造富有特色、带动性强、影响力大的农产品名牌。大力扶持农民专业合作经济组织，进一步加强农村经纪人队伍建设。实施科技入户工程，扩大专用玉米、优良种子的种植面积。控制重大动物疫病发生。继续搞好农业综合开发。搞好农业普查。

加强基础设施建设。加大农田水利基本建设力度，抓好防洪工程的除险加固。启动关山二库建设，整治堤防110公里，新建自来水267处、解决农村居民饮水安全问题，治理水土流失12万亩，新增黑色路面200公里、通油路行政村36个，抚顺县、新宾县基本实现村村通；开工建设南杂木至吉林梅河口高速公路，争取永陵至桓仁、辽宁中环线抚顺段两条高速公路开工建设。完成生物质气化工程10个和“一池三改”3500座，植树造林6万亩、封山育林30万亩。抓好新宾、清原两个县城，红透山、南杂木、永陵、石文4个国家级重点镇和草市省级中心镇，以及50个市级新农村试点村的建设，加快农村城镇化进程。开展农村面源污染治理，搞好畜牧业清洁生产和小城镇生活污水治理试点；引导农村劳动力向非农产业和城镇有序转移，多渠道增加农民收入。构建农村加快发展的动力机制。

（五）调整布局改善环境，大幅提升城市形象和价值

大规模拓展城市发展空间。加快沈抚“同城化”进程，推进资源共享，实现优势互补，提升区域竞争力。围绕抚沈高速、高山路和南环路建设，启动开发沿线经济带，向南北拓展城市发展的空间；借助百万吨乙烯和高新区的拉动，尽快建设东部新型工业区；依托抚顺经济开发区的迅猛发展态势和毗邻沈阳的区位优势，加快打造抚顺西部新城，从而构建更大规模的城市骨架，优化城市功能，增强承载能力。

大手笔高质量建设城市。一是调整城市布局。按照“人居向浑河沿岸集中、工业向外围园区集聚”的思路，鼓励新钢铁等工业企业搬出城内，进入园区，盘活部分“空壳”企业，利用腾空土地发展第三产业，支持危旧楼房及街路改造，通过改造，不仅拓展城市空间，而且改出一片新居、改出一片产业、改出一片景观。二是改造“两园”。启动以历史文化为主题的高尔山公园建设，完成以生态休闲为主题的劳动公园整体改造，打造高水准、大规模的城内风景区。三是完善交通网络。打通戈布通道，与沈吉高速公路连接；打通南环路，形成城市南环线，有效解决过境车辆穿越市区造成的交通拥堵问题。四是加大浑河支流治理力度。强化对浑河城市段支流的规划整治，力争在“十一五”末期全部建成景观河。五是绿化城市。植树100万株，新增绿地50公顷。六是建设一批民心工程。启动建设长途汽车中心客运站，加大城市主次干道、小街小巷、排水和“三供”管网的改造力度，整治居民小区环境100万平方米，亮化“摸黑”路20条，搞好棚户区排水和“三供”管网配套，建设棚改新区停车场7处。

大力度管理城市。积极构建“两级政府、三级管理、四级网络”的城市管理体系，充分发挥城区、街道、社区管理城市的主体作用，确保主次干道人工清扫保洁率100%、露天集贸市场整治率100%、生活垃圾清运率96%以上、污水处理量和处理天数均达到80%。坚持处罚和引导相结合，强化城市公共设施管理。从严整治，进一步改善交通秩序。立足搞活，积极稳妥推进公用事业单位投资主体多元化。加强监管，提高居民小区的物业管理水平。加大土地储备力度，拉动土地升值上市，提高公共收益。

高标准整治污染和地质灾害。加强污染防治，化学需氧量排放降低4%，确保浑河和大伙房水库等水体环境质量达到功能区标准；二氧化硫和烟粉尘排放量分别降低3.3%和2.4%，确保城区空气质量达到或好于二级标准的天数不少于290天，其中望花区不少于200天。强化矿山和城市环境综合整治，启动西露天矿北帮生态综合治理工程，综合整治因采煤引发的地质灾害，保护和改善地区生态环境。

（六）做好财税金融工作，增强服务经济社会能力

增加财政收入。坚持依法治税费，确保税收和非税收入应收尽收；加强财政债权清收、国有资产整合和改制企业国有股收益监管，严防政府资产流失；完善争取上级支持的激励政策，科学举债，为加快发展提供资金保障。

优化支出结构。按照公共财政要求，严格预算管理，确保支出按预算、追加按程序。强化财政资金的整合调度，加强对专项资金和项目资金的监管和审计，提高财政资金的使用效率和效益。牢固树立过紧日子和勤俭办事业的思想，杜绝铺张浪费，压缩一般性支出。加强对政府债务的整合和管理。

鼓励金融保险机构支持地方发展。加速社会诚信体系建设，优化地区金融环境。建立激励机制，搞好银企和项目对接，鼓励金融机构加大对地区发展的支持力度。加快保险业发展，充分发挥保险业的经济助推器和社会稳定器作用。

（七）推进文明建设，努力构建和谐抚顺

提升城乡文明程度。努力践行社会主义荣辱观，大力弘扬雷锋精神和开放、创新、敬业、奉献的城市精神，加强社会公德、职业道德和家庭美德教育，全面提高全民素质。普及科学知识，破除愚昧迷信，倡导文明健康的生活方式。以“窗口单位”为重点，大力开展文明创建活动，提高地区文明程度。

千方百计扩大就业再就业。以棚改回迁居民、零就业家庭为重点，做好就业、再就业工作，创业市场要在“五一”前全部开业。加大技能培训力度，增强下岗失业人员的就业能力。完成城镇实名制就业10万人以上，棚改回迁居民有就业能力和就业愿望的就业率92%以上，新增零就业家庭20日内实现至少1人就业，培训城镇劳动力2万人。

不断完善社会保障体系。以养老保险为重点，依法强力推进社会保险扩面和清欠，确保企业离退休人员养老金按时足额发放，各项保险基金基本步入良性循环。加强劳动合同管理，规范企业用工行为，切实维护劳动者的合法权益。做好被征地农民的补偿安置，探索失地农民的保障措施，保证进城务工人员工资支付，抓好农民工医疗、工伤保险工作。加大扶贫帮困力度，加强城乡低保动态管理，完善社会救助体系，切实保障困难群体的基本生活。积极探索城镇居民医疗保险体系建设。

继续推进棚户区改造。抓住省继续支持集中连片棚户区改造的机遇，加大向上争取力度，力争再用2年左右时间，基本完成列入省计划的棚户区改造任务。搞好棚改新区环境规划和建设。加快采沉区搬迁安置，全面完成新一期治理任务。

维护社会大局稳定。坚持“属地管理、分级负责”和“谁主管、谁负责”的原则，坚持市、县、乡三级联席会议制度，落实信访责任制和县区领导接待制度，实施双向责任追究，强化源头治理群体访，综合治理个人访；建立县区信访综合接待大厅，实行“一站式”接待，强化信访工作网络建设，确保实现省信访签状目标。坚持“打防结合，预防为主”方针，抓基层、打基础、苦练基本功，加强社会治安综合治理，保持严打高压态势，整合各类资源，构建覆盖城乡的治安巡防网络，严厉打击各种刑事犯罪和非法集资、传销活动，确保刑事发案率进一步下降、人民群众的安全感进一步增强，争创全省综合治理先进市。

抓细抓实抓好安全工作。加大安全监管和隐患整治力度，特别要加强对重点行业、重点单位和重点部位的检查和整治，关闭年产3万吨以下的小煤矿和不具备安全生产条件的企业，坚决遏制重特大事故发生。建立完善应急救援机制，提高对重大突发事件的快速反应和妥善处置能力。优化消费安全环境，坚持打假与扶优相结合，严厉打击制售假冒伪劣食品药品行为，保证人民群众健康安全。

（八）强力推进科技创新，协调发展各项社会事业

增强科技创新能力。积极推动企业与科研院所的交流合作，加强产学研合作平台、科技成果转化平台、科技服务平台、生产力促进中心和各级各类工程技术中心的建设，抓紧构建和完善以企业为主体的技术创新体系，重点推进15家企业与院校合作组建产学研战略联盟。加快高新区建设，打造国家级精细化工产业化基地，积极争取进入国家高新区行列。围绕产业和资源优势，抓好重大科技项目立项申报和达产达效，组织开展重点领域和重点技术的攻关，为地区发展提供强大动力。

强化人才教育工作。继续推进“人才强市”战略，抓住人才的培养、吸引和使用三个环节，着力建设政府机关、企业经营管理和专业技术三支人才队伍，营造人才辈出、人尽其才的社会氛围。坚持教育优先发展地位，继续加大对教育的投入，整合教育资源，促进各级各类教育健康发展，全面提高教育教学质量；以新一轮“普九”达标为重点，加强薄弱学校改造，加大向农村倾斜力度，努力实现义务教育均衡发展；大力发展职业教育，加快实训基地建设，继续推进校企合作，努力培养高素质的劳动者和技能型人才；完善扶困助学体系，确保学生不因贫困而失学。

积极发展文化卫生体育等项事业。大力开展健康文明、丰富多彩的群众性文化活动，全年广场演出2300场次以上，创造文化艺术精品；强化乡镇文化中心、村文化广场建设，建立健全农村文化网络；高标准完成平顶山遗址纪念馆的改造并投入使用。完善公共卫生和医疗救治体系，提高医疗救治水平；加强社区卫生服务体系建设，推进社区卫生纳入医保试点，逐步实现大病进医院、小病在社区；推进新型农村合作医疗，提高参合率、扩大受益面，改扩建乡镇卫生院19所。加强全民健身场所设施建设，广泛开展群众性体育健身活动，不断增强人民群众身体素质，努力提高竞技体育水平。巩固有线电视村村通成果，启动有线电视数字化整体平移工程。努力提高出生人口素质，人口出生率控制在5.85‰以内。切实维护妇女、儿童、老年人、残疾人的合法权益。全面落实党的民族宗教政策，切实维护民族团结。加强国防后备力量建设，争创全国双拥模范城。

推进节约型社会建设。把节约理念贯穿于生产、流通、消费和社会生活的各个领域，特别是大力发展节能、降耗、减污、增效的产业和项目，积极推广节能节水节材新工艺、新技术、新材料和新产品，大力发展循环经济，努力建设节约型机关、节约型单位、节约型企业。

（九）切实加强政府建设，不断提高工作水平

坚持勤政为民，建设服务型政府。推进政府机构改革和职能转变，抓好经济调节、市场监管，注重社会管理和公共服务，将政府资源更多地投向公共服务领域，提供更为丰富的公共产品。牢固树立以人为本的理念，多做群众急需的事，多做群众受益的事，多做打基础的事，多做起长远作用的事，使各项工作都能经得起实践、历史和群众的检验。全面落实《抚顺市优化经济发展环境条例》，严肃查处破坏软环境的人和事，创造公平、公正的地区发展环境。

坚持依法行政，建设, 法治型政府。自觉接受人大的依法监督，充分发挥政协和各民主党派、工商联、无党派人士、人民团体的民主监督和参政议政作用，主动听取各界意见。进一步落实国务院《全面推进依法行政实施纲要》，坚持把依法行政贯穿于政府决策、执行、监督的全过程，深入开展“五五”普法，不断提高立法质量，强化行政执法责任制。完善决策机制，坚持重大问题集体决策制度、专家咨询论证制度、社会公示和听证制度、决策责任制度，不断提高政府决策的科学化、民主化、法制化水平。

坚持改进作风，建设高效型政府。深化事业单位改革，全面实行首问负责制，减少审批事项，规范审批程序和行为，加强行政服务中心建设。进一步改进机关作风，坚持深入实际、深入基层、深入群众，讲真话、办实事、求实效。牢记“两个务必”，提倡勤俭节约，反对铺张浪费。积极推进政务公开，加强电子政务建设，降低行政成本，提高行政效率，使机关运转更加规范协调、办事更加公正透明、工作更加高效便民。健全目标考核监督体系，对行政不作为、影响行政效能、贻误行政工作等行为，严肃追究部门负责人和当事人的责任。

坚持标本兼治，建设廉洁型政府。认真贯彻执行领导干部廉洁自律的各项规定，全面落实党风廉政建设责任制和责任追究制，建立健全教育、制度、监督并重的预防和惩治腐败体系，加强审计监督，严肃查处各类腐败案件。突出抓好各级干部特别是领导干部的廉洁自律，突出抓好行政审批、政府采购、建设工程招投标等关键部位的制度完善和程序规范，注重从源头上惩治和预防腐败，树立政府廉洁从政的良好形象。

各位代表，今年是本届政府任期的最后一年。在抚顺发展的关键时期，人民的期望，不允许我们丝毫懈怠；时代的召唤，更要求我们奋发进取。让我们在市委的坚强领导下，在市人大和市政协的监督支持下，以更大的气魄、更高的热情、更实的作风，振奋精神，乘胜前进，为早日实现抚顺经济转型和全面振兴而努力奋斗!
